# Aging Well and Autism: A Narrative Review and Recommendations for Future Research

**DOI:** 10.3390/healthcare12121207

**Published:** 2024-06-17

**Authors:** Claire B. Klein, Laura G. Klinger

**Affiliations:** 1 Department of Psychology and Neuroscience, The University of North Carolina at Chapel Hill, Chapel Hill, NC 27599, USA; clairebklein@unc.edu; 2 UNC TEACCH Autism Program, Department of Psychiatry, The University of North Carolina at Chapel Hill, Chapel Hill, NC 27599, USA

**Keywords:** aging, autism, aging well, medical comorbidities, activities of daily living, cognitive functioning, physical functioning, mental health, social participation

## Abstract

With autism first recognized in the 1940s, the early cohorts of autistic children are beginning to enter older adulthood. Little is known about the experiences and outcomes of autistic older adults. In the general population, “successful aging” is a dominant model among gerontologists and is used to evaluate outcomes in older adulthood. This narrative review aims to provide a framework for understanding and supporting successful aging in older autistic adults. Using Fernández-Ballesteros’ four-domain model of “aging well” we review knowledge on aging and autism by examining outcomes in health and functioning, cognitive and physical functioning, positive affect and control, and social participation and engagement. Findings indicate that outcomes in autistic older adults are generally poor, marked by increased medical conditions, low adaptive skills, elevated risk of cognitive decline, limited physical activity, high rates of mental health conditions, low quality of life, and reduced social or community participation. Patterns of challenges are similar across cognitive abilities and profiles of autistic traits. Challenges and next steps in aging and autism research are identified, and future directions for the field are discussed.

## 1. Introduction

Autism is a lifelong, neurodevelopmental disability that emerges in early childhood and persists through adulthood [1]. Currently, 1 in 36 American children have a diagnosis of autism, marking an increase over the past twenty years, rising from estimates of 1 in 150 8-year-olds carrying an autism diagnosis in 2000 [2,3]. Based on these 2000 estimates, more than 274,000 autistic adults will enter their thirties in the United States in the coming years [3], and there could be many more autistic individuals diagnosed in adulthood or without a formal diagnosis [4]. This increase in autistic individuals transitioning to middle adulthood will continue to rise as younger generations of autistic individuals age. This narrative review aims to present current knowledge on aging in autistic adults through a developmental framework of aging well to make recommendations for future autism research and highlight potential service needs among middle-aged and older autistic adults.

A recent meta-analysis of longitudinal outcome studies of autism found that about half of adults in longitudinal studies were rated as having poor outcomes as measured by objective indicators, including employment, friendships, and independence/living situations [5]. Key predictors of outcome include lower IQ, diagnostic type, and diagnostic timing, with those receiving a diagnosis of childhood autism or infantile autism or those receiving a diagnosis longer ago experiencing poorer outcomes [5,6]. To date, little research has examined correlates of positive or “successful” aging. One study has applied Rowe and Kahn’s model of successful aging [7] to adults in the Australian Longitudinal Study of Adults with Autism (ALSAA). In this sample of autistic adults aged 40 and older, only 3.3% of the autistic adults met the criteria of successful aging, despite most of them having a diagnosis of Asperger’s and the cognitive abilities to complete a self-report form [7]. Historically, limited services were available to autistic individuals who now make up the aging population. For example, access to free, appropriate public education was not mandated in the United States until the Education for All Handicapped Children Act (later reauthorized as IDEA) in 1975, and autism was not covered under IDEA until 1990. It is well established that access to appropriate services early in life improves developmental trajectories [8,9]. For adults now in their 30s or 40s, access to early intervention services beginning in 1986 through Part H of IDEA, along with an improved understanding of autism diagnosis and therapies [10,11,12], has likely led to improved outcomes, while older generations of autistic adults were more likely to be institutionalized (those now age 70+) or to have no access to support in schools (those now age 60+). Over the past 80 years of autism research, changing diagnostic boundaries (see [13] for review), an increase in awareness of autism, and the development, dissemination, and implementation of support for autistic individuals have led to cohorts of autistic adults with a wide variety of profiles and experiences (i.e., cohort effects). Beyond this change in service access, contributing even further to these cohort effects is the challenge of the “lost generation” of autistic adults, a term coined by Lai and Baron-Cohen [4] to describe adults who may not have been identified or may not have met previous versions of diagnostic criteria but meet current DSM-5 criteria for autism.

The recent history of autism, changes in diagnostic boundaries, and associated cohort effects have compounded to create a dearth of information in the literature—we know very little about autism in middle and older adulthood. Even among cohorts that have been followed longitudinally, there is sparse information on autism in older adulthood. The average participants in our longitudinally followed cohorts were most recently studied in young adulthood (late 20s–30s [5]). However, many of these longitudinal cohorts have not been followed up on since the 1990s or early 2000s. They would now be in their 50s, 60s, and 70s, representing a significant gap in our knowledge of autism in older adulthood. On a positive note, research on adolescence, adulthood, and aging with autism is rapidly growing, with research on older autistics growing 392% since 2012, despite only making up 0.4% of the broader autism literature [14]. As the field continues to shift attention toward a lifespan research agenda, it will be important that researchers are aligned in terms of methodology, including establishing or confirming diagnoses in adults, integrating findings across diagnostic cohorts, differentiating between outcomes related to autism vs. co-occurring conditions, and drawing from the intellectual disability (ID) and gerontology fields to guide a path forward in autism.

## 2. Current Review

**Figure 1:**
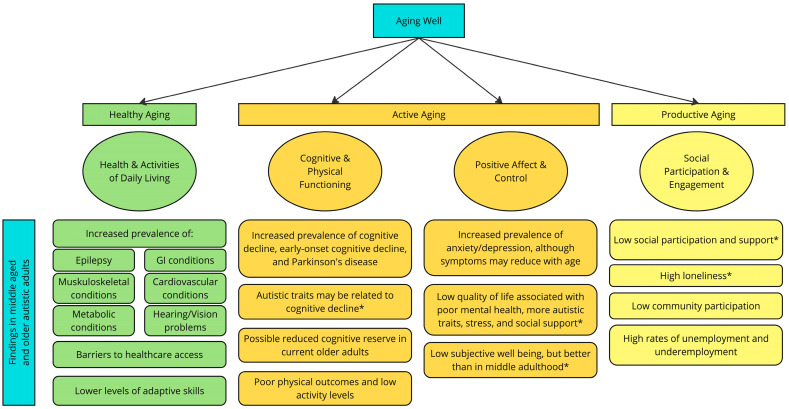
Aging well in autistic adults. Note. Figure adapted from [22]. * = Findings from research on autistic older adults without intellectual disability.

Building on previous reviews [15,16,17], findings will be presented through a multidimensional model of aging, integrating subjective and objective measures of biomedical aspects and psychosocial factors contributing to outcomes in older adulthood [18]. In 2008, Fernández-Ballesteros and colleagues integrated dominant theories of aging, successful aging (high cognitive and physical function, engagement in life, and avoiding disease and disability; [19]) and aging well (healthy aging, active aging, and productive aging), to propose a four-domain model of aging well in the general population (see Figure 1; [20]). The four domains include health and functioning, cognitive and physical functioning, affect and control, and social participation [20,21,22]. The current review will examine outcomes for autistic adults in the four domains of aging well, highlighting gaps in the literature, implications for support needs, and recommendations for future research. In considering each domain, aging will be viewed as a developmental experience that begins before adults enter what is typically considered “older age”. The primary focus of this work will be on research including autistic older adults (using the World Health Organization definition of age 60 years and older; [23]) but findings from late middle adulthood (age 50 years and over, as recommended by [24]) with implications for future work in the field of aging and autism will be included as well. Due to limitations in the field on conducting research on autistic older adults and few studies on this topic (described above), a broad range of samples will be included in the review, including studies on individuals with a confirmed autism diagnosis at any time in their life, individuals self-reporting a clinical diagnosis of autism from a healthcare professional, and individuals reporting high autistic traits (without a confirmed clinical diagnosis). To clarify the generalizability of findings from the wide variety of inclusion criteria used in adult autism research, each study’s sample will be described within the review.

### 2.1. Health and Activities of Daily Living

One common definition of healthy aging is the absence of disease and disability [19]. In the four-domain model of aging well, this definition is broadened to include the ability to continue daily living activities without functional limitations and maintain good health [22]. In the general population, physical health is predictive of disability in activities of daily living among older adults [25,26]. Together, health and functioning describe the component of maintaining a level of health that allows for participation in daily activities.

Co-occurring physical health problems are common in autistic adults (See Table 1 for a summary). In one healthcare system study of autistic adults ages 18–65+ (19% with co-occurring ID), chronic medical conditions were more common in autistic adults than in a non-autistic control group, including conditions known to frequently occur in autistic children, such as GI conditions, sleep disorders, nutrition conditions, and metabolic disorders [27]. Conditions related to aging with increased risk in autistic adults included cardiovascular diseases (present in 37% of autistic adults) and neurologic diseases (present in 39% of autistic adults; [27]). The increased odds of co-occurring medical conditions were consistent when autistic older adults (ages 65+, 44% with co-occurring ID) were examined in a larger sample of Medicare data from the United States, with higher prevalence rates across almost all conditions, and the largest differences between autistic and non-autistic adults in epilepsy and GI conditions, even when controlling for demographic variables [28]. Among conditions common in older adults, odds among autistic adults ranged from 1.4–3.1 for metabolic conditions, 1.6–2.1 for cardiovascular conditions, and 1.6–4.4 for musculoskeletal conditions [28]. When examining whether medical conditions changed across the adult lifespan through a comparison of older and younger autistic adults without ID from the Simons Powering Autism Research Knowledge (SPARK) database (mean age = 33.4, range 18–85 years), the only age difference in the endorsement of medical conditions was that adults over the age of 60 had more hearing or vision problems [29]. This suggests that co-occurring health conditions are elevated in autistic adults across the adult lifespan, although the types of medical conditions examined were less comprehensive than other studies using healthcare claims data. Autistic older adults with ID may be at even greater risk of experiencing co-occurring health conditions. When comparing rates of medical conditions between autistic older adults with and without ID enrolled in Medicare (ages 65+), those with co-occurring ID had the largest increased odds of epilepsy (43%), osteoporosis (23%), GI conditions (60%), respiratory infections (38%), and thyroid disorders (36% of autistic adults with ID; [30]). A similar pattern of elevated occurrence of medical conditions was found in a naturalistic study of autistic adults (mean age = 49.9, range 30–70+ years, 84% with ID) receiving care from a community agency. Among those over the age of 50 (*n* = 40), 18% had a seizure disorder, 59% had a GI condition, and 28% had hypertension [31]. 

The use of administrative healthcare data to identify common conditions in autistic older adults leverages the strengths of large datasets but also has limitations with regard to characterizing the sample, including specificity of diagnosis (e.g., timing of autism diagnosis, ID status), differences in methodology regarding diagnostic codes for study inclusion (e.g., requiring one outpatient autism code [28,30], versus more than one [27]), and a lack of validation of diagnostic coding for autistic adults [32]. Despite these limitations, the combination of recent healthcare claims data research provides consistent cross-sectional findings suggesting that autistic individuals, especially those with ID, carry an increased risk of physical health conditions across their lifespans and may require a higher level of ongoing management and care for these conditions compared with non-autistic adults.

Potential reasons for these increased health conditions include possible etiologic or genetic predisposition to health conditions (e.g., epilepsy; [33]), lifestyle factors common in autism (e.g., exercise, sleep, diet; [34]), reduced access to preventive healthcare associated with characteristics of autism (e.g., social communication differences, challenges with the sensory environment, executive function challenges; [35]), and risks associated with long term polypharmacy (e.g., antipsychotic medications; [36]). Future research is needed to examine the temporal development of health conditions [37] and if changes to modifiable risk factors (e.g., lifestyle factors, changes to pharmacological approaches, training for health care providers) are effective in promoting better health outcomes in autistic older adults. For example, research examining whether better sleep quality during adolescence and early adulthood leads to fewer medical conditions during older adulthood would help address the question of whether a change in lifestyle could result in better physical health outcomes in this population.

Although research on the intersection of health conditions and functional outcomes in autistic older adults is limited, the connection between health and daily living skills has been described in studies of general aging [25,26], in ID research [38], and in qualitative work with autistic middle-aged adults and their caregivers [39]. Autistic adults often have lower levels of adaptive skills that remain low throughout their life course [40,41,42]. In one study of middle-aged and older autistic adults with high support needs (mean age = 49.9, range 30–70+, 84% with ID), better overall health was associated with independence in basic activities of daily living [43]. In this cross-sectional approach with mostly adults with co-occurring ID, independence in daily activities was not associated with age [43], indicating that daily living skills may remain stable in later adulthood, although longitudinal research with autistic adults with and without ID found that gains in adaptive behavior continue in middle and older adulthood, but are slowed (mean age = 35.47, range = 20–58, 63% with ID; [44]).

Several processes may influence daily living skills. Aging-related changes in executive function can lead to impairment of daily living skills in the general older population [45], and executive function skills are a known challenge and barrier to daily living skills in autistic adults [46,47]. Future research is needed to continue to characterize daily living skill difficulties and needs in autistic older adults. For example, increased perceived stress is a predictor of daily living skills in middle-aged and older adults without ID from SPARK research match (mean age = 38.47, range 18–83 years, [48]). Future work is needed to examine therapeutic approaches used in older adults to maintain daily living skills (e.g., using modifications to maximize person–environment fit) and whether they are effective in autistic older adults in preserving daily living skills and improving physical health.

### 2.2. Positive Affect and Control

Fernández-Ballesteros [22] defines affect and control as having good psychological functioning, good life satisfaction, coping skills (control), and purpose. In older adults in the general population, psychological outcomes are variable, with individuals showing stable, worsening, or improving trajectories of depressive symptoms [49,50]. Psychosocial determinants of health are associated with improving trajectories of depressive symptoms (e.g., education level, stressful events, social engagement; [49,50]). Other areas of this domain, including life satisfaction, overlap with quality of life [51]. In older adults, quality of life tends to increase in late middle age and peak at 68 years before gradually decreasing [52]. Influences on quality of life in the general population of older adults include daily living skills, social relationships, and financial resources [52]. In autism research in older adulthood, areas of positive affect and control include psychological functioning (i.e., mental health symptoms) and quality of life as the dominant measures of life satisfaction.

It is well-established that mental health outcomes in autistic adults are often poor (See Table 2 for Summary; [53,54]). In a meta-analysis of the prevalence of anxiety and depression in autistic individuals across adulthood (capturing studies ranging from late adolescence to older adulthood), the estimated current prevalence rates in the full sample were 27% for anxiety and 23% for depression [53]. Estimates of autistic adults with ID were lower, with current and lifetime prevalence estimates of 20% for anxiety and 14% for depression, a significant difference in depression rates compared to autistic adults without ID (26%; [53]). Across groups, these rates are much higher than estimates in the general population (e.g., prevalence estimates of anxiety between 1 and 12% and of depression at 7%; [55,56]). In a meta-analysis of studies from childhood through adulthood, depression prevalence rates were higher in samples with autistic adults with higher cognitive abilities and with higher proportions of white participants [54]. Research on middle and older age autistic adults diagnosed in childhood (mean age = 43, range 29–64 years; mean IQ in adulthood 69.7, IQ range 20–139) found a similar rate of depression (25%) in comparison to the non-ID prevalence rate seen in the meta-analysis, but a lower rate of anxiety (14%; [57]). Lifetime rates of psychiatric conditions are high, with a rate of 67.8% reported in older adults with autistic traits (mean age = 62.97, range 50–81 years; [58]) and 66.7% in autistic older adults (mean age = 63.9, age range 55–79 years; [59]).

A limitation of these meta-analyses is that due to the age ranges in the studies, they could not examine associations between age and psychiatric symptoms. In research on mental health outcomes in autistic middle-aged and older adults with ID, challenging behaviors that may be related to anxiety and depression have been observed to decrease over a period of 20–35 years (mean age = 49.9, range 30–70+ years, 84% with ID; [31]) and occur less often in older compared to younger adults with behavioral characteristics of autism (mean age = 44.5, range 18–90 years, 100% with ID; [60]). In studies comparing rates of psychiatric conditions in younger and older autistic adults without ID (pooled age range 18–85 years) from three studies, with one using an autism research registry (SPARK, [29]), one including autistic adults mostly diagnosed in childhood [59], and one including autistic adults mostly diagnosed in adulthood [61], a pattern of the highest rates occurring in middle adulthood and lower ratings of psychiatric conditions in older adulthood emerged. Research in adults with high autistic traits has found a similar pattern of older age being associated with fewer psychiatric conditions in the autistic trait and control groups [58]. Only one study, a follow-up of autistic adults diagnosed as children (mean age = 43, range 29–64 years), did not find associations between psychiatric conditions and age [57]. However, one risk factor for lifetime psychiatric diagnoses and higher rates of mental health conditions in adulthood is receiving a late autism diagnosis (e.g., after age 21; [29]), which may contribute to these findings. Together, these studies indicate a consistent finding of elevated rates of psychiatric conditions in autistic older adults across cognitive abilities, although a trend toward symptom reduction with age was identified in longitudinal and cross-sectional research.

Quality of life, capturing satisfaction in physical, psychological, social, and environmental outcomes, contributes to positive affect and control and is a frequently assessed construct in autism research (see [62] for a review). Autistic middle-aged and older adults without ID report low quality of life compared to normative outcomes [48,61,63,64,65,66,67]. This finding appears to be consistent across samples and inclusion criteria. A lower quality of life has been reported in samples of middle-aged and older adults including autistic adults from the SPARK research match (mean age = 52.19, range 40–83, [64]; mean age = 38.47, range 18–83 years, [48]), autistic adults with a clinical diagnosis of autism (mean age = 44.1, range 19–80 years; [63]), autistic adults from the Adult Autism Spectrum Cohort-UK (ASC-UK) diagnosed in adulthood (mean age =41.61, range 17–80 years; [65]), and autistic adults diagnosed across age ranges (mean age = 44.96, range 21–71 years; [61]). Two studies only included autistic older adults over the age of 50, one from ASC-UK diagnosed on average at age 56 (mean age = 61.5, range not reported; [67]) and adults with an autism diagnosis or a high score on an autism screener (mean age = 63.7, range 53–83 years; [66]). 

Contributions to this lower quality of life in autistic middle-aged and older adults include poor mental health outcomes [61,63,65,67], more autistic traits [63,65], elevated perceived stress [48], and low subjective social support ([64], discussed in Social Participation and Engagement). In autistic older adults, quality of life has not been found to be related to normative outcome ratings (e.g., employment, independent living, and social engagement; [67]) although more research is needed to confirm this finding. Associations between age and quality of life were either non-significant or very small [63,64,66] except for mixed reports on whether the social quality of life is reduced [65] or improved [61] in older age. For autistic adults with ID, initial findings in older adulthood indicate similar quality of life outcomes for those with ID with and without autism features [60].

Broadly, findings indicate that older age may lead to a reduction in mental health symptoms but does not lead to improvements in quality of life in autistic adults. Quality of life appears to remain low and stable through older adulthood. There is significant evidence to support that poor mental health outcomes are a contributing factor to these poor quality of life outcomes. Future research is needed on subjective well-being in autistic older adults, as this may be an outcome that improves with older age, examining whether quality of life can be improved with changes in normative adult outcomes (e.g., employment), and examining quality of life in autistic older adults with ID. To better support autistic older adults, we need more knowledge on mental health interventions’ efficacy in older adulthood and possible cascading benefits in quality of life in older adulthood.

### 2.3. Social Participation and Engagement

Among older adults in the general population, indicators of high social participation and engagement include maintaining networks of social support and active engagement with other people and their community, such as through work or volunteering [22]. Community participation builds social cohesion and support, demonstrating the interrelatedness of concepts within this domain [68]. Loneliness, reported by 25–29% of older adults in the US, is related to mental and physical health outcomes in the general population of older adults [69]. The effects of social relationships on physical and mental health can be positive and negative, and their effects on health may be even more potent than physical determinants of health (e.g., diabetes; [70]).

Given the social–communication challenges often associated with autism, it is not surprising that autistic adults report less social participation than adults in the general population. Social support in autistic middle-aged and older adults is associated with quality of life [64] and reduced perceived stress [71]. In research with middle-aged and older autistic adults without ID from the SPARK research match (mean age = 52.19, range 40–83 years), perceived social support, but not social interactions, predicted all aspects of quality of life [64]. Across adulthood, social support and interactions remained low and stable in this cross-sectional study [64]. In a study examining similar constructs of interpersonal support and loneliness, autistic adults self-reporting a clinical autism diagnosis (mean age = 40.8, range 19–73 years, 85.8% diagnosed in adulthood) were more likely to report greater loneliness and less social support than non-autistic adults [71]. Loneliness was an indicator of poor mental health, and both social support and loneliness were associated with the level of perceived stress [71]. In a study of subjective well-being in younger and older autistic adults with a clinical diagnosis of autism (mean age = 44.1, range 19–80 years), low subjective well-being was primarily due to a lack of achievement, isolation from the community and others, and concerns about one’s health and future prospects [63].

One reason for reports of loneliness and low social engagement among autistic adults may be lower community participation rates, examined through a recent systematic review ([72]; however, only 6% of the studies had a mean age of 40 years or older). Autistic middle-aged and older adults may participate less and in fewer activities, have less satisfaction with activities, and reduce participation in older age or as depressive symptoms increase [60,73]. Comparisons of adults with ID with autism characteristics to individuals with ID only (mean age = 44.5, range 18–90 years) indicated that low levels of adaptive skills among the autistic adults accounted for reduced social participation, including less variety in activities and less time participating in activities [60]. In a comparative study between autistic and non-autistic adults from the ALSAA cohort (mean age = 42.7, range 25–85 years), autistic adults reported lower satisfaction with leisure activities [73]. Indicators of reduced participation and poor leisure outcomes included older age and more depressive symptoms [73]. Future research is needed to continue to examine barriers and facilitators of community participation, the benefits of participation, and how to support authentic participation by autistic older adults with and without ID.

Currently, there are also many barriers to autistic adults living a community-integrated life, especially in terms of living options. In qualitative research with autistic middle-aged and older autistic adults without ID (mean age = 40, range 27–53 years) and caregivers of autistic middle-aged and older adults, community living emerged as a critical part of adults’ and their caregivers’ conceptualization of independence [39]. Caregivers reported concerns about planning for the future and who will support their children when they are gone. Caregivers and autistic adults reported problems with wait lists for group homes, difficulty finding the right fit, low quality of options, and cost. General safety in the community was also highlighted as a concern of caregivers and a barrier to community-integrated living [39].

Opportunities for productivity (e.g., employment) are often limited for autistic adults and may be intertwined with social participation. Autistic adults report low rates of employment and underemployment in young adulthood [74]. Outcomes from a 40-year longitudinal study in the UK of autistic adults diagnosed in childhood (mean age = 43.4 years, range 29–64 years, all without ID in childhood) found that most adults were in sheltered employment or unemployed (69%) or in unskilled jobs (16%; [57]). In a sample of older adults in the UK diagnosed late in adulthood (mean age of diagnosis = 56.4 years, SD = 7.15; 1.4% ID), 36% of adults were unemployed, volunteering, or in supported employment [67]. These findings highlight that across cognitive ability and age of diagnosis, employment outcomes are often limited among autistic older adults and are likely related to social participation and engagement.

Despite this growing knowledge base, little is known about how to support autistic middle-aged and older autistic adults in social or community engagement. The potential benefits of commonly used interventions to support participation in younger adulthood (e.g., social groups) have not yet been examined in older adulthood. Studies of the benefits of employment or volunteering on social participation and related quality of life have not yet been conducted in older autistic adults. Future research on support specifically targeting older adults is needed, and future work will benefit from asking autistic older adults what kinds or amounts of social participation are important to them as they age, as has been done with non-autistic older adults [75,76].

### 2.4. Cognitive and Physical Functioning

The final component of Fernandez-Ballesteros’ four-domain model of aging well is cognitive and physical functioning. Cognitive functioning is defined as maintaining cognitive abilities during aging and compensating for cognitive changes [77], a strategy associated with daily functioning and independence in non-autistic older adults [78]. In a recent review of meta-analyses examining risk factors for dementia, the most robust evidence indicated risks of use of benzodiazepines, depression, and low frequency of social contact [79]. Other modifiable risk factors such as education level, intellectually engaging employment, and engagement in leisure activities did not have strong support in this meta-analysis, although previous research has highlighted these as important for maintaining cognitive functioning into older adulthood [80,81,82]. A recent meta-analysis examining the association between physical activity and aging found that the long-term benefits of physical activity were strongest in middle adulthood [83], with benefits including lower rates of illness, cognitive impairment, and mental health conditions in addition to less need for daily living supports [83]. Overall, cognitive and physical functioning describe areas with age-related changes, but some evidence suggests they may be able to be preserved or compensated for to improve outcomes.

Recent research has established that autistic adults are at increased risk of experiencing cognitive decline and that the onset of this cognitive decline may occur earlier than in the general population [84,85]. Autistic adults enrolled in Medicaid in the United States had a higher prevalence of early-onset dementia (i.e., before age 65; 4.04%) when compared to non-autistic adults (0.97%), with the prevalence rising for adults with both autism and ID (5.22%; [85]). In a recent study with a large sample of middle-aged and older adults from SPARK research match, 30% of autistic adults (mean age = 55.62, all without ID) reported experiences of cognitive decline [84]. Higher autistic traits were associated with screening positive for cognitive decline [84]. In a large-scale study of older adults without ID comparing those reporting high vs. low autistic traits (not diagnostic groups; mean age = 61, range 50–80 years), those reporting high autistic traits were more likely to report cognitive decline, although controlling for depression (a risk factor for cognitive decline; [79]) attenuated this finding [86]. In work examining autistic traits in non-autistic adults with dementia, elevated autistic trait ratings were indicative of earlier-onset and more severe cognitive impairment [87]. Together, these samples and research studies complement each other to describe an increased risk for dementia and cognitive decline in autistic adults across cognitive abilities, and that autistic traits may be related to cognitive decline across autistic and non-autistic middle-aged and older adults. Other outcomes in autistic older adults, including low education levels [88], unemployment or underemployment (described in Social Participation and Engagement), depression (described in Positive Affect and Control), risks associated with medications (e.g., anticholinergic cognitive burden), and low levels of physical activity [89] may be contributing to increased risk of experiencing cognitive decline in autistic adults [79,81,90], although more longitudinal research is needed to examine predictors and modifiable indicators of cognitive decline and cognitive reserve in autistic adults from mid to older adulthood.

Cognitive research in middle-aged and older autistic adults has revealed some preliminary evidence for more heterogeneous cognitive profiles [91]. Some studies have reported atypical trajectories of age-related cognitive decline in autistic adults compared to non-autistic adults, suggesting autism-specific declines [92], although other studies have identified parallel trajectories of cognitive change, suggesting cognitive aging trajectories consistent with aging in the general population [93,94,95]. Variability in findings may be due to cross-sectional designs, small sample sizes (n ranges = 23–88 autistic adults), differences in measurement (e.g., different approaches to measuring executive function), recruitment differences (e.g., community recruitment vs. recruitment from clinical centers), differences across countries, and variability in autism diagnoses, although each study used common autism diagnostic measures to confirm diagnosis and none of the studies included individuals with ID (IQ range across studies = 81–153; [92,93,94,95]). In one longitudinal study of cognitive aging with a larger sample of autistic adults (*n* = 128; baseline mean age = 52.2, range 24–79 years) without ID mostly diagnosed in adulthood, no evidence for accelerated decline was observed across measures of memory, executive function, or response speed [96]. Together, these findings indicate a growing evidence base supporting similar trajectories of age-related cognitive changes among autistic and non-autistic middle-aged and older adults with average or higher cognitive abilities, although more research is needed as this is not necessarily consistent with previous research discussing early onset of conditions associated with cognitive decline. Future work is needed with autistic individuals representative of the full spectrum of autistic older adults, including those with ID.

In addition to cognitive decline, studies using healthcare records, neurological assessments, and surveys consistently indicate that autistic adults may be at greater risk of experiencing specific neurological conditions associated with aging, including Parkinson’s Disease. Using healthcare records, autistic adults enrolled in Medicare in the United States were reported to have a 6.1× greater risk of parkinsonism compared to a population sample of enrollees [28]. Compared to a prevalence rate of 0.006% in the general population aged 45 and older [97], a rate of 32% was found in a combined sample of autistic older adults diagnosed using neurological assessment (mean ages = 57 and 51.2, range 40–77 years, 73% with an IQ below 50), suggesting not only a higher rate but also an earlier onset of parkinsonism [98]. In a large sample of autistic adults in the Netherlands and the US without ID, 17–33% of autistic adults screened positive using a self-report measure of motor features related to parkinsonism (mean ages = 58/59, range 50–83 years, [99]). Rates on a screener from this non-ID self-report sample are much higher than estimates from healthcare claims including autistic adults with and without ID (6.6%; [28,30]). However, the estimates using a screener or assessment were more similar despite being from samples of autistic adults without ID (17–33%; [99]) and autistic adults with ID (32%; [98]). Research in the ID field indicates that ID increases the risk of parkinsonism (OR = 2.82 between ID and the general population; [100]) with a possible cumulative effect with ID and autism (OR = 1.24–1.3 between ASD+ID and ASD only; [30,101]). Together, these studies suggest that there is a higher co-occurrence of Parkinson’s disease among autistic adults compared to the general population, which is not specific to those with co-occurring ID. However, future research needs to disentangle risks associated with neuroleptic medications from risks associated with autism and ID status to understand the prevalence of the condition.

Autistic older adults may also be at risk of experiencing poor physical functioning outcomes (e.g., sleep, diet, exercise). Although most research on physical outcomes has focused on medical conditions (e.g., obesity, diabetes, hypertension, and arthritis described in Health and Functioning), autistic adults may also exhibit poor sleep, diet, and exercise, and accelerated physical aging in general [34,102]. In a sample of autistic adults self-reporting an autism diagnosis (mean age = 41, range 16–90 years, <2% reporting ID), autistic adults reported that they exercised less, had worse eating habits, and were more likely to have sleep challenges than a non-autistic control group [34]. In autistic adults with ID (mean age = 25.79, range 18–55 years), research found adults were likely to be overweight or obese (60.5%), have poor motor skills (36.8%), and engage in minimal physical activity (only 10.4% classified as active or highly active; [89]). However, age differences in these outcomes were not examined, and it is unclear how age-related changes interact with physical outcomes in autism. Other research on physical outcomes in older adulthood has been in samples of adults reporting high autistic traits. In a birth cohort study examining the association between self-reported autistic traits and the pace of aging (measured by a decline in physical biomarkers) among 45-year-olds in New Zealand, higher autistic trait ratings were associated with a faster pace of aging above and beyond socioeconomic status and cognitive abilities, capturing early decline from age 26–45 years [35]. Although it is a limitation of this study that they examined individuals with high autistic traits, this is the first large-scale study to indicate that an accelerated pace of physical aging may be related to autism. Across all measures of cognitive functioning (cognitive decline, age-related neurological conditions, and physical aging), research suggests that autistic adults with and without co-occurring ID may be at risk of early onset and higher rates of cognitive decline than the general population.

## 3. Discussion

This review aimed to present current knowledge of aging in autistic adults through Fernandez-Ballesteros’ four-domain model of aging well to make recommendations for future autism research and highlight potential service needs among middle-aged and older autistic adults. This review reinforces and expands on Hwang and colleagues’ [7] report that many autistic older adults are not aging well. Outcomes among autistic older adults indicate increased medical conditions and low adaptive skills (Health and Activities of Daily Living), elevated risk of cognitive decline and poor physical outcomes characterized by low activity levels (Cognitive and Physical Functioning), high rates of mental health conditions and low quality of life (Positive Affect and Control), and limited social participation, elevated loneliness, and high unemployment/underemployment rates (Social Participation and Engagement; Figure 1). Findings consistently indicate an increased likelihood of poor outcomes, regardless of cognitive level, although knowledge gaps exist. Much of our current understanding of autistic older adults is from self-report research with adults without ID, many of whom were diagnosed in late adulthood or reported high autistic traits without a formal autism diagnosis. These older adults likely had different experiences than those with ID or childhood diagnoses, although researchers have not yet examined differences in adult outcomes between those diagnosed in childhood or adulthood or considered the impact of cohort effects on outcomes. These findings that many autistic adults, even those with the ability to self-report, are not aging well, create a call to action with regards to the need to identify services to support a different developmental trajectory through aging.

### 3.1. Challenges and Limitations of Current Research

The wide variety of samples characterized throughout this review provides insight into why cohort effects may be a barrier to our understanding of aging and autism. First, relatively few longitudinal cohorts are being followed in older adulthood (e.g., Australian Longitudinal Study of Adults with Autism [ALSAA], d’arc initiative, The Ageing with Autism Project, Adult Autism Spectrum Cohort-UK; [24]), with many of the findings reported in this review coming from the same samples of autistic adults. Other studies have primarily relied on self-report data from individuals without ID (e.g., SPARK) or used medical billing codes to determine diagnosis (e.g., Medicare, Medicaid). The implication of this is that no single cohort or sample is “representative” of the broad spectrum of abilities, experiences, and outcomes captured under a diagnosis of autism. Further, these samples of autistic adults and related findings may be impacted by other biases, including ascertainment bias in identifying autistic individuals (e.g., an underrepresentation in research of autistic individuals with ID; [103]) and healthy survivor effects [104,105,106]. Autistic individuals diagnosed in adulthood are likely over-represented in aging research, with some samples reporting a mean age of diagnosis as late as in their 50s (e.g., [67]). Previous research on sampling bias in autism research has also highlighted limitations in recruitment strategies, such as social media, a common method in adult research [107]. However, it is not yet known if phenotypic differences exist between individuals diagnosed in childhood or adulthood. Jadav and Bal [29] provided an initial comparison of co-occurring conditions between those diagnosed before or after age 21 (6.1% with ID), identifying an increased prevalence of psychiatric conditions among those diagnosed as adults.

### 3.2. Recommendations for Research on Aging with Autism

In future research with longitudinal cohorts, it will be important to use measurement tools developed for autistic adults that capture the full spectrum of autism [108], and to harmonize measures across studies. Future work to characterize similarities and potential differences (e.g., levels or areas of support needs) between diagnostic cohorts (e.g., decade of diagnosis/DSM version, childhood vs. adulthood) could inform our understanding of heterogeneous outcomes in older adulthood. These methods may help anticipate support needs and improve planning for older autistic adults. The majority of studies have been cross-sectional due to the challenges involved in studying development across the lifespan with longitudinal methods. One promising method not yet leveraged in autism aging research is accelerated longitudinal design, which provides an approach for examining both cross-sectional and longitudinal changes across development over a short period of time.

#### 3.2.1. Recommendations for Measurement of Aging-Related Conditions

A key challenge for research on cognitive and aging-related conditions in autistic adults with and without ID is a lack of measures validated in samples of autistic adults. Previous research in cognitively able adults has used self and informant-report screeners of cognitive decline (e.g., the AD8, the IQCode-SF; [84,86,109,110]), with one study demonstrating convergent validity with a measure of memory problems [84]. However, future work is needed to continue to validate these screeners along with performance-based indicators of cognitive impairment (e.g., the Montreal Cognitive Assessment, MoCA; [111]), which have been used in autistic adults [92,112]. For adults with ID, a systematic review and evaluation of measures recommended screeners of cognitive decline/dementia that have primarily been developed for adults with Down Syndrome [113], except for the Dementia Screening Questionnaire for Individuals with Intellectual Disabilities (DSQIID; [114]), although the DSQIID has not been specifically validated for *autistic* adults with ID. In research on parkinsonism, autism research to date has utilized a self-report screener (Parkinsonism Screening Questionnaire, PSQ; [115]) and a clinical rating scale (Unified Parkinson’s Disease Rating Scale, UPDRS; [116]), although modifications were made to the rating scale to accommodate comprehension differences in the sample of autistic adults with ID [98]. As research and practice in this area continue to expand and a need for valid and reliable measurement tools grows, future work should focus on continuing to evaluate the performance of these measures in autistic adults and studying potential modifications to the measures. 

#### 3.2.2. Recommendations for Adult Diagnosis

Issues of ascertainment are related to challenges in adult diagnosis. Centers for Disease Control and Prevention (CDC) surveillance systems in the United States only capture individuals born in 1992 and later, yet the estimated prevalence rate of autism in adulthood is 2.21% [117]. Autistic adults often receive other diagnoses before getting an autism diagnosis (see [118] for a review of experiences and characteristics of adults receiving an adult diagnosis). The recommended minimum for autism assessment is for a clinician with expertise in autism to integrate developmental history from an informant, medical history, psychiatric symptoms, cognitive testing, adaptive skills, and standardized behavioral observation (i.e., the ADOS; [119]). Although there is no current consensus on adult assessment guidelines [120], recommendations for clinicians exist [4,121]. They emphasize pre-assessment activities, including individualizing the process and having a team member with expertise in mental health diagnosis [121]. In carrying out the assessment, recommended methods include consideration of sensitivity and specificity of self-report measures, awareness of generational differences in informants reporting autistic traits, and acknowledgment of the presence of learned compensatory social strategies or camouflaging [4]. When family members are not available to provide the developmental history, other informants may include other relatives, teachers, or community members who knew the adult when they were young (see [4] for an overview). Other sources of information to aid diagnosis may include school records, videotapes, or childhood documents. As research on autistic older adults grows, researchers may find it interesting to consider the previous autism diagnoses an adult would have qualified for if diagnosed in childhood, allowing for comparisons or sub-setting based on the diagnosis.

#### 3.2.3. Recommendations for Reporting Ascertainment

Characterizing samples clearly and accurately is essential to extending the implications of findings to appropriate groups within the autism spectrum. Not all autistic older adults will have the same support needs, benefit from similar interventions, or face the same aging-related trajectories. In 2019, an autism and aging special interest group recommended aligning background questions, measures of autistic traits, and measures of quality of life to harmonize information [24]. The recommendation included collecting information on the age of diagnosis, reported in many of the studies included in this review. Recommendations and examples of reporting sample ascertainment observed throughout this review included a records review to confirm that adults met DSM-5 criteria for autism (e.g., [31]), reporting proportions of original diagnoses received (e.g., autistic disorder, Asperger’s, PDD-NOS, infantile autism; [59,73]), mean age and range of first diagnosis, and percentage of the individuals who received an adult diagnosis (e.g., [65]). There is currently no agreed-upon cutoff of what defines an adult diagnosis of autism, although two studies have categorized adult diagnosis as occurring after the age of 21 [29,122], which in the US would indicate leaving the school system without an autism diagnosis. Language and characterization requirements may vary between journals, editors, and reviewers (e.g., differences in language using the same ascertainment; [60,123], but it is important to comprehensively describe strengths and limitations for generalizability.

#### 3.2.4. Recommendations for Services

Results show that autistic adults will need services to support health-related conditions, daily living skills, mental health, and community participation. As the number of children diagnosed with autism rises, the field is seeing a parallel increase in the number of autistic individuals entering middle and older adulthood. Compounding this transition to middle adulthood is a lack of services available for autistic adults throughout adulthood following high school exit, commonly referred to as the “services cliff” for young autistic adults [124]. A service delivery system to support aging well in this population is clearly needed. Autistic adults and stakeholders have called for work to build our understanding of how to care for autistic older adults, including community-based support, housing, healthy aging programs, and support in planning for the future [125,126,127]. Aging caregivers have shared concerns about who will care for their loved ones when they can no longer care for them [39,126]. The current interagency autism coordinating committee (IACC) in the United States recommends research on increasing support for autistic individuals throughout their lifespan, with specific attention to enhancing existing services and support networks for autistic older adults [128]. Currently, little is known about what interventions are appropriate or effective for autistic older adults. For example, the utility of therapies for anxiety and depression (e.g., Cognitive Behavioral Therapy, Dialectical Behavioral Therapy, and Mindfulness approaches) has been examined in samples including autistic adults [127,129,130] but has not yet been specifically examined in older autistic adults. Research is also needed to examine psychoeducational interventions for older adults [131] and self-care practices [132] as avenues for intervention with older autistic adults.

In 2019, ref. [133] called for more work to bridge research, practice, and policy between the aging and intellectual/developmental disabilities (IDD) fields and common areas of need. Proposed areas of intervention overlap included family caregiving research, an area of need in autism research. Examples of adapted interventions from aging for IDD included health promotion interventions, dementia care, and fall prevention programs [133], but these types of interventions or physical activity programs have yet to be studied in autism [127,134]. However, research and practice recommendations for supporting older autistic adults in residential care have been developed [135], community participation programs across ages for autistic adults with ID are emerging [136], and self-care practices in older autistic adults have been described [132]. Future work may continue to examine supports for non-autistic older adults (e.g., the use of visual supports for dementia care; [137,138]) and in individuals with ID (e.g., behavioral activation; [139]) for use with older autistic adults. While much of the work on autism and aging has been conducted in the United States, there is growing research being conducted across the world, including the UK, Netherlands, and Australia, suggesting that the need to focus on autism in older age is a global health issue. As interventions and policies to support autistic adults are developed and implemented, it will be important to continue to share best practices globally.

### 3.3. Limitations of the Current Review

Due to the limited availability of knowledge on autistic older adults, this work used available literature to define aging outcomes rather than specific measures. Future research is needed to continue to examine the trajectories of each domain of aging well and how they interact. Knowledge of how these domains affect one another is emerging (e.g., the observed connection between stress and activities of daily living [48]), but temporal relationships, such as how decline in one domain impacts others, are not yet known. For instance, in the general population, a decline in executive functioning (Cognitive and Physical Functioning) may impact adaptive skills (Health and Activities of Daily Living; [45]) but it is unclear if this connection is true for older autistic adults. This information will help inform healthy aging programs to preserve abilities in each area, potentially building resilience to the effects of aging in autistic older adults through increased knowledge and awareness of age-related changes, improved health communication, and a focus on maintaining skills.

This work was also limited by using a model of aging well based on the general population. Research is needed to ensure that these metrics indeed represent aging well in older autistic adults. While preventing cognitive decline is a clear goal across diagnoses, it is less clear if social participation represents the same goal for those with and without an autism diagnosis. Alternative multidimensional models have been proposed, taking a more continuous approach to measurement and allowing compensation in one domain from others [140], capturing the dynamic process of providing environmental support in areas of need [133], or emphasizing modifiable health outcomes (e.g., regularly engaging in physical activity) rather than common chronic illnesses (e.g., epilepsy; [141]). It is our hope that this review will spur research that examines whether this model of aging well from the general population is appropriate for examining aging well in autistic adults. Future research through these approaches may explore how autistic older adults cope, compensate, and show resilience to age-related changes.

## 4. Conclusions

This review suggests that many autistic adults, even those with good cognitive and communication skills, are not experiencing aging well. Across diverse samples, the body of current research indicates that outcomes for autistic older adults are generally poor, marked by increased medical conditions, low adaptive skills, elevated risk of cognitive decline, limited physical activity, high rates of mental health conditions, low quality of life, and reduced social or community participation. Patterns of challenges are similar across cognitive abilities and profiles of autistic traits, although our knowledge of aging in autistic adults with co-occurring ID is more limited. As our knowledge of autism in adulthood continues to grow, it remains important to recognize that older adulthood is the culmination of experiences throughout the lifespan. Cohort effects and diagnostic timing have likely influenced these experiences. Challenges for research and practice in the field of autism and aging include a need for valid measurement tools for aging-related conditions, the complexities of autism diagnosis in older adulthood, and challenges with ascertainment. From a services perspective, the transition to older adulthood is another critical transition period in autism, particularly when the support of parents is no longer available. This transition necessitates the same level of tailored support provided during earlier transition periods (e.g., early childhood to elementary school and high school to adulthood). Urgent attention is required to develop appropriate services for this high-needs group and expand service system capacity as the population of autistic older adults continues to increase. As we continue to conduct research on autistic older adults and develop tailored supports for aging, it is important to include the perspectives of autistic individuals and caregivers in the development of research priorities and supports.

## Figures and Tables

**Table 1:** Prevalence rates of physical health conditions in current research.

Study	Data/Recruitment Source	Sample Size (Full Autism Sample)	Age Range (Full Age Range)	Co-Occurring ID (Full Autism Sample)	Comparison Group	Condition	Prevalence	Odds Ratio
Croen et al., 2015 [27]	Kaiser Permanente Medical Care Program in Northern California	1507	18–65+	19.2%	Non-autistic adults	GI disorders	34.70%	1.35 (1.16–1.57) ^a^
						Diabetes	7.56%	2.18 (1.62–2.93) ^a^
						Thyroid disease	7.03%	2.46 (1.81–3.33) ^a^
						Cardiovascular diseases	36.96%	2.54 (2.13–3.02) ^a^
						Neurologic diseases	39.28%	2.21 (1.90–2.57) ^a^
						Nutrition conditions	37.23%	2.68 (2.29–3.12) ^a^
						Sleep disorders	17.58%	1.92 (1.58–2.33) ^a^
						Hearing impairment	4.71%	2.35 (1.63–3.38) ^a^
Hand et al., 2020 [28]	Medicare	4685	65+	43.80%	Matched population comparison group	Upper or lower GI disorders	48.60%	2.7 (2.5–2.9) ^b^
						Diabetes	36.60%	1.6 (1.5–1.7) ^b^
						Thyroid disorders	31.70%	3.1 (2.9–3.3) ^b^
						Hypertension	66.50%	2.0 (1.9–2.2) ^b^
						Heart disease	54.20%	2.1 (2.0–2.3) ^b^
						Epilepsy	26.40%	18.9 (17.2–20.7) ^b^
						Osteoporosis	16.60%	4.4 (4.0–4.8) ^b^
Jadav et al., 2022 [29]	SPARK Database	174 (4657)	60+ (18–85)	0%	-	Deafness/hearing loss	14.40%	-
						Cataract	13.80%	-
						Seizure disorder or epilepsy	4.60%	-
						Sleep disorder	37.40%	-
Gilmore et al., 2021 [30]	Medicare	2054 (4685)	65+	100% (44%)	Autistic adults without ID	Upper or lower GI disorders	53.40%	1.5 (1.3–1.7) ^c^
						Other GI conditions	59.90%	2.0 (1.8–2.2) ^c^
						Thyroid disorders	36.10%	1.5 (1.3–1.7) ^c^
						Epilepsy	42.60%	4.5 (3.9–5.2) ^c^
						Osteoporosis	23.40%	2.7 (2.3–3.2) ^c^
						Respiratory Infections	38.20%	1.8 (1.6-2.1) ^c^
Wise et al., 2017 [31]	Adults receiving care from a community agency	40 (74)	50–70+	83% (84%)	-	GI Disorder	59.00%	-
						Hypertension	28.20%	-
						Seizure Disorder	17.90%	-
						Cardiovascular disease	7.70%	-

Selected conditions based on categories of conditions examined in each study, overlap with other studies, relatedness to aging, and odds ratios. ^a^ Compared to non-autistic adults, 99% confidence interval adjusted for sex, age, and race/ethnicity. ^b^ Compared to non-autistic adults, 95% confidence interval adjusted for sex, age, race/ethnicity, rural residence, and estimated household income. ^c^ Compared to autistic adults without ID, 95% confidence interval adjusted for sex, age, race/ethnicity, rural residence, estimated household income, and duration of observation.

**Table 2:** Prevalence rates of mental health conditions in current research.

Study	Data/Recruitment Source	Sample Size (Full Autism Sample)	Age Range (Full Age Range)	ID Status	Condition	Current Prevalence (95% CI)	Lifetime Prevalence (95% CI)
Hollocks et al., 2019 [53]	Meta-analysis	Range 1444–25,714	16–84	Full Sample	Any anxiety	27% (17–37%)	42% (35–50%)
					Depression	23% (17–29%)	37% (27–47%)
				Non-ID	Any anxiety	24% (19–43%)	-
					Depression	26% (20–32%)	-
				ID	Any anxiety	20% (7–39%)	-
					Depression	14% (5–28%)	-
Hudson et al., 2019 [54]	Meta-analysis	NR	18+ (<18–18+)	ID and non-ID	Depression	19.4% (9.2–36.5%)	40.2% (22.8–60.6%)
Moss et al., 2015 [57]	Individuals seen as children between 1950 and 1979	58	29–64	IQ range 20–139	Anxiety	-	14% ^a^
					Depression	-	25% ^a^
Stewart et al., 2021 [58]	PROTECT study in the UK	276	50–81	Non-ID	Any psychiatric diagnosis	-	67.80%
Lever & Geurts, 2016 [59]	Mental health institutions and client organization websites	45 (138)	55–79 (19–79)	Non-ID	Any psychiatric disorder	-	66.70%
					Anxiety	-	42.20%
					Depression	-	42.20%

Reported prevalence rates focused on anxiety, depression, and overall estimates of psychiatric disorders for comparison across studies. NR = Not reported. ^a^ Occurrence since the age of 16 years.

## Data Availability

Not applicable.
